# Glutathione *S*-Transferase Alpha 4 Prevents Dopamine Neurodegeneration in a Rat Alpha-Synuclein Model of Parkinson’s Disease

**DOI:** 10.3389/fneur.2018.00222

**Published:** 2018-04-06

**Authors:** Michael Jewett, Elna Dickson, Kajsa Brolin, Matilde Negrini, Itzia Jimenez-Ferrer, Maria Swanberg

**Affiliations:** Translational Neurogenetics Unit, Department of Experimental Medical Science, Wallenberg Neuroscience Center, Lund University, Lund, Sweden

**Keywords:** Parkinson’s disease, α-synuclein, dopaminergic neurons, neuroprotection, glutathione *S*-transferase alpha 4, Vra1

## Abstract

Parkinson’s disease (PD) is a common, progressive neurodegenerative disease, which typically presents itself with a range of motor symptoms, like resting tremor, bradykinesia, and rigidity, but also non-motor symptoms such as fatigue, constipation, and sleep disturbance. Neuropathologically, PD is characterized by loss of dopaminergic cells in the substantia nigra pars compacta (SNpc) and Lewy bodies, neuronal inclusions containing α-synuclein (α-syn). Mutations and copy number variations of *SNCA*, the gene encoding α-syn, are linked to familial PD and common *SNCA* gene variants are associated to idiopathic PD. Large-scale genome-wide association studies have identified risk variants across another 40 loci associated to idiopathic PD. These risk variants do not, however, explain all the genetic contribution to idiopathic PD. The rat *Vra1* locus has been linked to neuroprotection after nerve- and brain injury in rats. *Vra1* includes the glutathione *S*-transferase alpha 4 (*Gsta4*) gene, which encodes a protein involved in clearing lipid peroxidation by-products. The DA.VRA1 congenic rat strain, carrying PVG alleles in *Vra1* on a DA strain background, was recently reported to express higher levels of *Gsta4* transcripts and to display partial neuroprotection of SNpc dopaminergic neurons in a 6-hydroxydopamine (6-OHDA) induced model for PD. Since α-syn expression increases the risk for PD in a dose-dependent manner, we assessed the neuroprotective effects of *Vra1* in an α-syn-induced PD model. Human wild-type α-syn was overexpressed by unilateral injections of the rAAV6-α-syn vector in the SNpc of DA and DA.VRA1 congenic rats. *Gsta4* gene expression levels were significantly higher in the striatum and midbrain of DA.VRA1 compared to DA rats at 3 weeks post surgery, in both the ipsilateral and contralateral sides. At 8 weeks post surgery, DA.VRA1 rats suffered significantly lower fiber loss in the striatum and lower loss of dopaminergic neurons in the SNpc compared to DA. Immunofluorescent stainings showed co-expression of Gsta4 with Gfap at 8 weeks suggesting that astrocytic expression of Gsta4 underlies *Vra1*-mediated neuroprotection to α-syn induced pathology. This is the second PD model in which *Vra1* is linked to protection of the nigrostriatal pathway, solidifying Gsta4 as a potential therapeutic target in PD.

## Introduction

Parkinson’s disease (PD) is a progressive neurodegenerative disease characterized by loss of dopaminergic neurons in the substantia nigra pars compacta (SNpc) resulting in a range of motor and non-motor symptoms. One of the pathological hallmarks of PD is the accumulation of α-synuclein (α-syn) protein, which is abundant in neuronal inclusions termed Lewy bodies and Lewy neurites (1). About 10% of PD cases are familial, and so far, mutations in seven genes have been linked to PD with a recessive or dominant inheritance pattern (2). The remaining 90% are classified as idiopathic PD with a complex etiology, meaning that both genetic and environmental factors contribute to the disease (3, 4). So far, 41 PD risk loci have been confirmed as associated to idiopathic PD (5, 6). There is, however, still a substantial missing heritability, i.e., undiscovered genetic risk factors contributing to PD etiology.

The *Vra1* region on rat chromosome 8 was linked to neuroprotection after ventral root avulsion (VRA) was performed in an intercross between the inbred Dark Agouti (DA) and Piebald Virol Glaxo (PVG.1AV1) rat strains (7). The congenic DA.VRA1 strain, carrying PVG.1AV1 alleles in the neuroprotective *Vra1* region on a DA strain background, was used to fine map *Vra1* and several candidate genes were discovered (8). Glutathione *S-*transferase alpha 4 (Gsta4), a protein involved in the elimination of lipid peroxidation by-products, such as 4-hydroxy-2-nonenal (HNE) (9), was subsequently identified as the strongest candidate gene regulating neurodegeneration in response to VRA (10) and traumatic brain injury in DA.VRA1 congenic rats (11).

Glutathione S-transferase alpha 4 belongs to the alpha class of glutathione *S-*transferases (GSTs). GSTs are a family of isoenzymes involved in cellular detoxification mechanisms including clearance of lipid peroxidation by-products through glutathione (GSH) conjugation (9, 12). Not much is known about the expression patterns of Gsta4 in humans or in rodents, although studies suggest that it is expressed ubiquitously (13, 14). Furthermore, while rat Gsta4 is only 60% homologous with human GSTA4, the two enzymes have similar catalytic affinity to HNE (9), making it a valuable experimental target. Genetic associations have been made between GSTA4 mutations and risk for certain types of cancer (15, 16), but not much is known about the role of GSTA4 in PD. However, HNE has been shown to be significantly elevated in PD brains (17–19), suggesting that GSTA4 is somehow affected and could be a key player in the disease. In order to study the effects of Gsta4 in a PD model that induces high levels of oxidative stress, we recently performed unilateral striatal 6-hydroxydopamine (6-OHDA) lesions in DA and DA.VRA1 rats. At 8 weeks post lesion, DA.VRA1 congenic rats suffered less striatal fiber loss and were more resistant to SNpc neuronal cell death compared to DA rats. In addition, *Gsta4* expression was elevated in the striatum and midbrain of DA.VRA1 rats at 2 days post lesion compared to DA, which is when the first signs of the degenerative process occur after 6-OHDA injections (20), but stabilized already after 7 days. This suggests that *Gsta4* plays a major role in protecting DA.VRA1 rats from a dopaminergic-specific toxin and that it exerts its effects early in the degenerative process (21). The 6-OHDA lesion, however, does not model the α-syn pathology seen in PD.

The genetics linking α-syn to PD is abundant. Mutations in *SNCA* encoding α-syn are linked to monogenic PD (22), and copy-number variation of SNCA is linked to PD in a dose-dependent manner with several duplications (23–32) and triplications (32–34) being reported. In addition, common variants of *SNCA* are associated to idiopathic PD (35). Thus α-syn is clearly implicated in PD etiology and is, therefore, widely used in PD animal models: from transgenic rodent models (36) to viral vector-mediated models (37), with the latter being able to deliver a more consistent and progressive PD-like phenotype (38).

It has been shown that the overexpression of α-syn in rodents through the use of viral vectors leads to a progressive pathology with loss of midbrain dopaminergic neurons (39, 40). In fact, reports have shown that recombinant adeno-associated viral (rAAV) vector-mediated overexpression of α-syn in rats reproduces several of the neuropathological aspects seen in patients (41–43), making it a relevant research model for studying PD. There is also evidence that α-syn activates oxidative stress mechanisms; for example, studies have shown that α-syn overexpression, like 6-OHDA, leads to mitochondrial impairment, which in turn leads to the production of reactive oxygen species (ROS) and lipid peroxidation (44–47).

In this study, we investigated if the *Vra1* locus encoding *Gsta4* mediates neuroprotection after overexpression of human wildtype (WT) α-syn in the rat SNpc. Compared to DA, DA.VRA1 congenic rats displayed higher gene expression levels of *Gsta4* in the striatum and SNpc at 3 weeks after α-syn overexpression. Furthermore, at 8 weeks after α-syn overexpression, we observed less degeneration of dopaminergic fibers in the striatum and their respective cell bodies in the SNpc. Similar to what was previously reported from the 6-OHDA model (21), Gsta4 was expressed in astrocytes in the SNpc at 8 weeks post rAAV injections. These results suggest that the *Vra1* locus protects from α-syn-induced PD-like neurodegeneration and that astrocytes mediate this action through expression of Gsta4.

## Materials and Methods

### Research Model

For this study, we used two different inbred strains of rats: Dark Agouti (DA) and DA.VRA1, a congenic strain developed by transferring Vra1 alleles from the PVG^av1^ strain to a DA background strains as previously described (21). 64 male rats were used in this study (33 DA and 31 DA.VRA1 congenics), weighing approximately 220–250 g. Professor Piehl at the Karolinska Institutet, Stockholm, Sweden generously provided the founders for each strain. 51 (28 DA and 23 DA.VRA1) animals were subjected to unilateral injections of an rAAV6 vector construct to overexpress human WT α-syn, while 13 (5 DA and 8 DA.VRA1) were injected with the same vector construct to overexpress GFP in the midbrain at 12 weeks of age with the following titers: α-syn (1.2E + 14 gc/ml) and GFP (3.2E + 14 gc/ml). The expression of both transgenes is led by the synapsin-1 promoter and enhanced with the woodchuck hepatitis virus posttranscriptional regulatory element (WPRE) (42). For quantification of dopaminergic neurodgeneration, the rAAV6-GFP-injected animals of both strains were pooled together as one group and abbreviated DA (GFP). This was done because no differences were found between the two strains after O.D. measurements in the striatum and stereological measurements in the SNpc (see Quantification of Dopaminergic Fiber Loss in the Striatum and Quantification of Dopaminergic Cell Loss in SNpc). The rats were given *ad libitum* access to food and water during a 12 h light/dark cycle and housed 2–3 per cage. 32 animals were sacrificed at 3 weeks post surgery for gene expression and immunofluorescence analysis, while 32 others were sacrificed at 8 weeks post surgery for histological analysis. All procedures described were approved by the Ethical Committee for the use of laboratory animals in the Lund/Malmö region.

### Surgical Procedure

All surgical procedures were performed as described previously (21). 3 µl of rAAV6-α-syn or -GFP were unilaterally injected in the SNpc, which was targeting using the following coordinates, given in millimeters relative to bregma and dural surface (48): AP = −5.3, ML = −1.7, DV = −7.2. After the procedure, 0.15 ml Metacam (Apoteksbolaget, Sweden) was injected s.c. for postoperative analgesia. All animals were then placed in clean cages on a heated pad for recovery.

### Tissue Preparation and Histology

Most tissue preparation and immunostainings were performed as described previously (21) For DAB stainings in this study, the following primary antibodies were used: mouse anti-tyrosine hydroxylase (TH) (1:1,000, Immunostar, Hudson, WI, USA), rabbit anti-vesicular monoamine transporter 2 (VMAT2) (1:4,000, Immunostar Hudson, WI USA), mouse anti-human WT α-syn (1:2,000, Santa Cruz, CA, USA), and chicken anti-GFP (1:20,000 Abcam, Cambridge, UK). The SNpc sections were given an initial antigen-retrieval incubation in Tris/EDTA (pH 9.0) at 80°C for 45 min when stained for TH.

Double immunofluorescence stainings were performed as described previously (21). The primary antibodies used were rabbit anti-GSTA4 (1:100 Antibodies-online GmbH, Aachen, Germany), mouse anti-Gfap (1:1,000, Santa Cruz, CA USA), chicken anti-IBA1 (1:500 Synaptic Systems, Göttingen, Germany), and mouse anti-NeuN (1:1,000 Millipore, Billerica, MA USA) and were incubated together at 4°C. To compare immunofluorescent stainings of midbrain and striatum for Gsta4 and Gfap at 3 and 8 weeks, stainings were performed in parallel and images were taken with the same settings. All images were captured at high-resolution with the confocal Leica SP8 microscope (Leica Microsystems, Wetzlar, Germany).

### Quantification of Dopaminergic Fiber Loss in the Striatum

Striatum pictures were acquired as described previously (21). Dorsal (D) striatal TH^+^ fiber density was evaluated as optical density (O.D.) by image densitometry at six coronal levels (+1.60, +1.15, +0.70, +0.25, −0.20, −0.75 mm from bregma) using the ImageJ software (https://imagej.nih.gov NIH, USA). The Rodbard calibration function within the software was used to normalize the range of gray-scale (0–255) into O.D. values. Each image was transformed into 8-bit (gray-scale). The contralateral (CL) and ipsilateral (IL) striatum was delineated for each section, and the O.D. values representing the strength of the TH^+^ staining from each side were obtained. O.D. values from the corpus callosum were used to correct for non-specific background staining. Finally, the dopaminergic fiber loss was expressed as relative to the CL side versus the intact side for each animal. Three DA rats were excluded from the analysis due to complications during surgery or with tissue processing, leaving 7 DA, and 6 DA.VRA1 for quantification. Striatum divisions between D and ventral (V) are shown in Figure 2B.

### Quantification of Dopaminergic Cell Loss in SNpc

Dopaminergic neurons in the SNpc were quantified by stereology of TH^+^ cells according to the optical fractionator principle using the Stereo Investigator software (MBF Bioscience, USA) as described previously (21). With a Leitz DMRBE microscope (Leica, Germany), a 5× objective was used to delineate the areas of interest for each section, and a 100× oil-immersion objective was used for the cell counting. A frame ratio of 11% was assigned to each slide, and the average mounted section thickness (h) was 24.3 µm (±2.1). The average number of dopaminergic neurons counted in each individual was 286 (±73). A maximal Gundersen coefficient of error (C.E.) (49) of 0.08 was accepted. The counting criteria used matches the one previously used (21). Three animals were excluded from the analysis due to complications during surgery or with tissue processing, leaving 7 DA, and 6 DA.VRA1 for quantification.

### Gene Expression Analysis

Animals were sedated and sacrificed at 3 weeks postsurgery as described previously (21). Pieces of right and left striatum and ventral midbrain weighing approximately 30 mg were dissected from the brain and placed in lysing matrix beaded tubes (MP Biomedicals, USA) and immediately stored at −80°C. The RNeasy Mini kit (Qiagen, Germany) was used to extract RNA from these samples, following the supplier’s protocol with some variations already mentioned in Jewett et al. (21). Reverse Transcription and Quantitative (RT)-PCR followed using the SuperScript^®^ III First-Strand Synthesis System (Invitrogen, USA) and SSoAdvanced Universal SYBR green Supermix (BioRad, USA), respectively. qPCR was performed with this protocol: 5 µl Supermix + 0.5 µl of each primer + 4 µl cDNA for each sample. Sample amplification followed this 3-step protocol (1. 30 s at 95°C; 2. 60 s at 62°C for 39 cycles; 3. 5 min at 68°C) with the following primers (5′-3′): *Gsta4* (fw: GACCGTCCTGAAGTTCTAGTGA, rev: TGCCTCTGGAATGCTCTGT), *gapdh* (fw: CAACTCCCTCAAGATTGTCAGCAA, rev: GGCATGGACTGTGGTCATGA) and β*-actin* (fw: AAGTCCCTCACCCTCCCAAAAG, rev: AAGCAATGCTGTCACCTTCCC). Levels of *Gsta4* gene expression were calculated using 2^−ΔΔCq^ (50) and normalized relating each value to CL DA of within the respective brain regions (striatum and SNpc).

### Statistical Analysis

All statistics were performed with GraphPad Prism (version 7, La Jolla, CA, USA). Values are expressed as mean ± SD. Due to the low number of animals used for each data set, a Shapiro–Wilk normality test was performed to determine whether to proceed with parametric or non-parametric tests. Stereology and densitometry differences between groups were analyzed using a one-way ANOVA followed by Bonferroni’s multiple comparisons *post hoc* test; statistical significance was set at *p-*value < 0.05. Correlation analysis was performed using the Pearson correlation coefficient (*r*), statistical significance was set at *p-*value <0.05, and a 95% confidence interval was used. A one-way ANOVA was used to calculate gene expression differences between groups at each time point, followed by Bonferroni’s multiple comparisons *post hoc* test.

## Results

### DA.VRA1 Rats Present Higher Levels of Gsta4 Gene Expression

**Figure 1:**
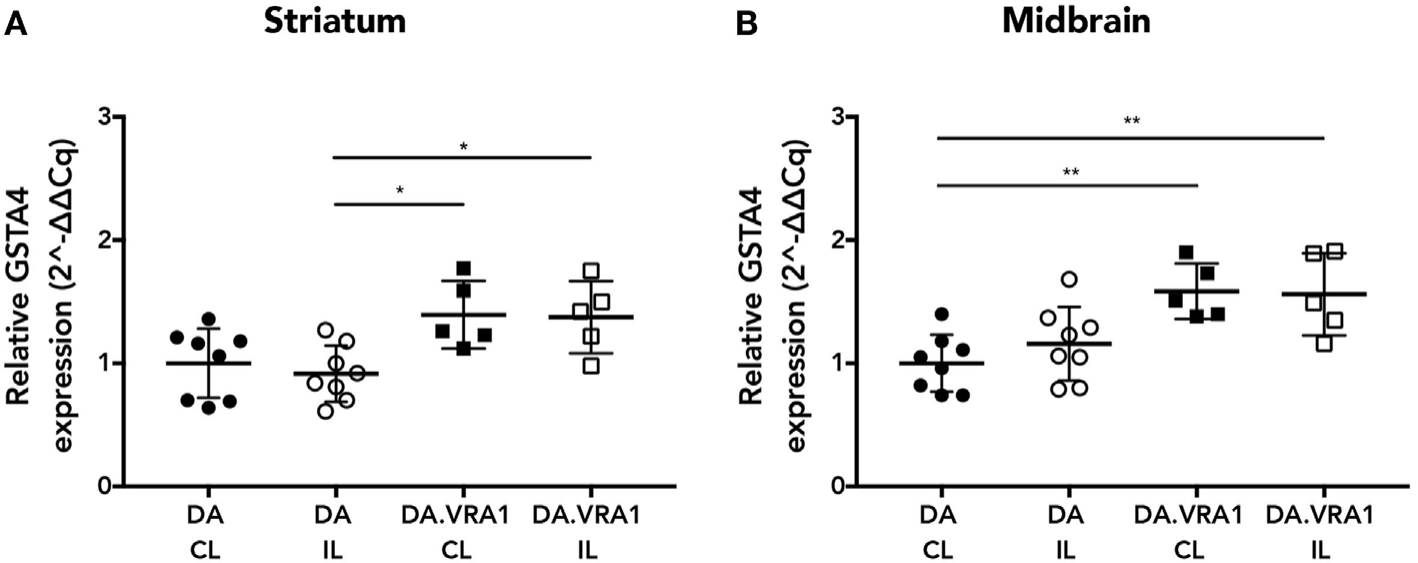
Glutathione *S*-transferase alpha 4 (*Gsta4*) gene expression in the striatum and midbrain after recombinant adeno-associated viral (rAAV)-mediated α-syn overexpression in the substantia nigra pars compacta. *Gsta4* expression was significantly higher in DA.VRA1 compared to DA striatum **(A)** and midbrain **(B)** at 3 weeks post rAAV injection. There was no difference within each strain between ipsilateral (IL) and contralateral (CL) sides. Data were normalized to DA CL mean values for the respective brain region. Mean ± SD, *p < 0.05, **p < 0.01.

Glutathione *S*-transferase alpha 4 has been shown to be upregulated in IL and CL sides of both striatum and midbrain of DA and DA.VRA1 rats at 2 days post striatal 6-OHDA injections, which is when the first signs of neuronal degeneration become evident within that model (20, 21). For this study, we wanted to investigate *Gsta4* expression levels within those same regions at a time point relevant to dopaminergic degeneration within the model of nigral rAAV-α-syn overexpression. We, therefore, chose to assess gene expression of *Gsta4* at 3 weeks after rAAV-mediated α-syn injections in the SNpc using the CL striatum and midbrain regions as internal controls (42). *Gsta4* expression is significantly higher in the striatum (Figure 1A) (*p* < 0.05) and midbrain (Figure 1B) (*p* < 0.01) of DA.VRA1 compared to DA rats. There are no differences in *Gsta4* expression between the CL and IL side within each strain (Figures 1A,B).

### DA.VRA1 Congenic Rats Display Less Dopaminergic Fiber Loss After α-Syn Overexpression

**Figure 2:**
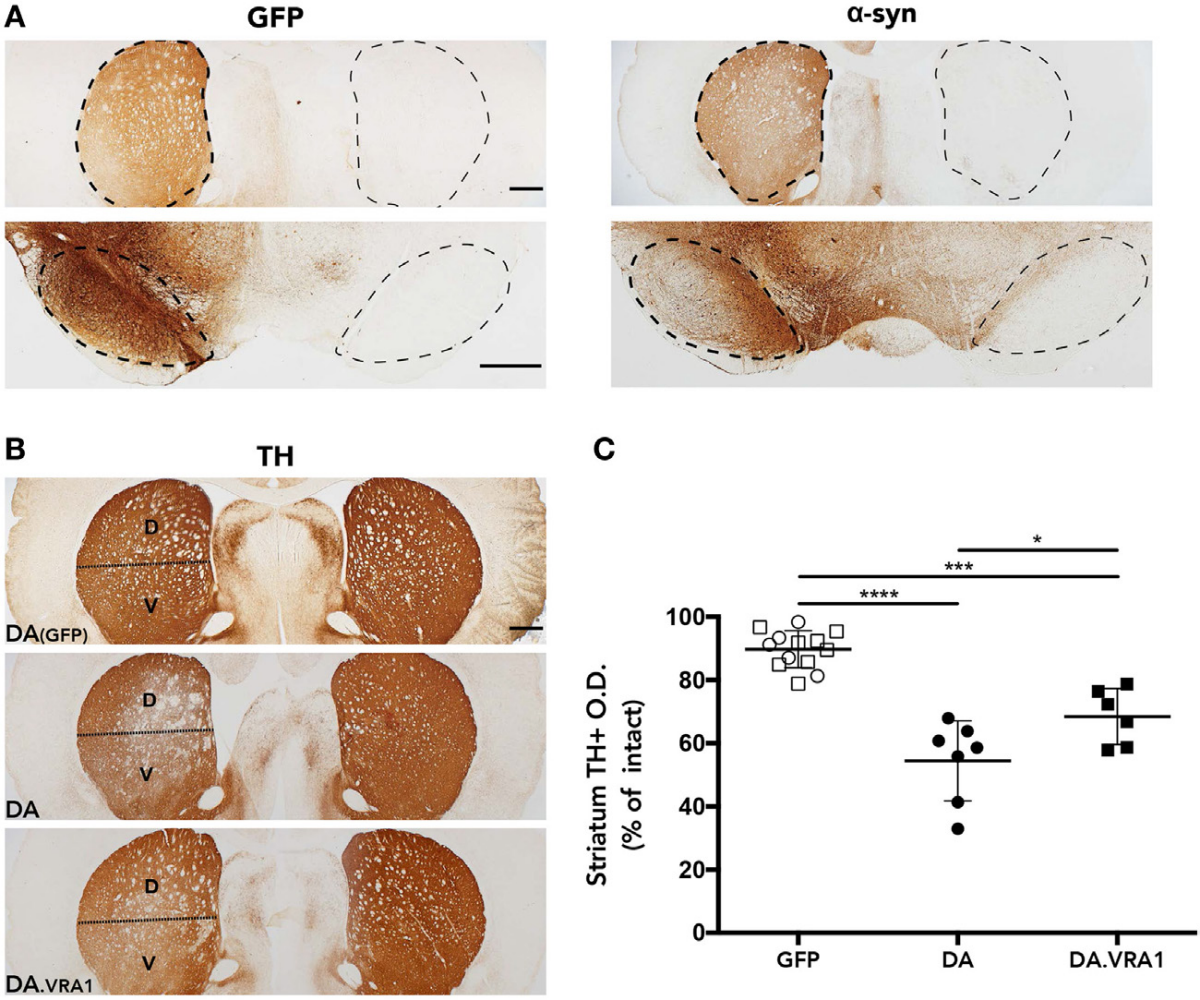
Striatal dopaminergic fibers are protected from α-syn-induced degeneration by alleles in the *Vra1* locus. **(A)** Sample images showing GFP/α-syn transgene expression in the striatum and midbrain 8 weeks post unilateral recombinant adeno-associated viral (rAAV)-GFP/α-syn injections into the substantia nigra pars compacta (SNpc). **(B)** Representative pictures from DA (GFP), DA, and DA.VRA1 rats showing dopaminergic fibers in the striatum stained for tyrosine hydroxylase (TH) at 8 weeks after rAAV-α-syn injection. The lesioned striatum is divided in two parts: dorsal (D), the region receiving most afferent projections from the cells of the SNpc, and ventral (V). **(C)** Optical density quantification of TH^+^ fibers in the lesioned relative to intact dorsal striatum at 8 weeks post surgery. DA.VRA1 rats display higher levels of remaining TH^+^ fibers in the lesioned striatum compared to DA. Mean ± SD, *p* < 0.05 based on a one-way ANOVA followed by a Bonferroni *post hoc* test. O.D., optical density, scale bars = 500 µm.

The rAAV-α-syn model was chosen because it has been shown to produce partial and progressive degeneration of dopaminergic fibers in the striatum and cell bodies in the SNpc, a hallmark of PD (42). In order to evaluate accurate targeting and expression of the transgenes, striatum and midbrain sections were stained for GFP and human WT α-syn. The histological analysis shows high levels of both GFP and α-syn expression with accurate targeting of the nigrostriatal pathway (Figure 2A). Furthermore, our stainings of dopaminergic (TH^+^) fibers in the striatum indicate that mainly the dorsal striatum was denervated upon α-syn overexpression. Therefore, the striatum was subdivided into dorsal, mainly innervated by the SN, and ventral, mainly innervated by the ventral tegmental area (51) (Figure 2B). Optical densitometry measuring the density of TH^+^ fibers of the IL compared to the CL striatum points to a higher proportion of remaining TH^+^ fibers in the IL dorsal striatum of DA.VRA1 compared to DA rats [mean (SD): 69 (13) vs. 54 (9)%, *p* < 0.023], with DA(GFP) animals being unaffected (Figure 2C). PVG.1AV1 alleles in the *Vra1* locus thus protected striatal dopaminergic fibers of DA.VRA1 congenic rats from α-syn-induced degeneration.

### DA.VRA1 Congenic Rats Are Partially Protected From Dopaminergic Cell Loss in SNpc

**Figure 3:**
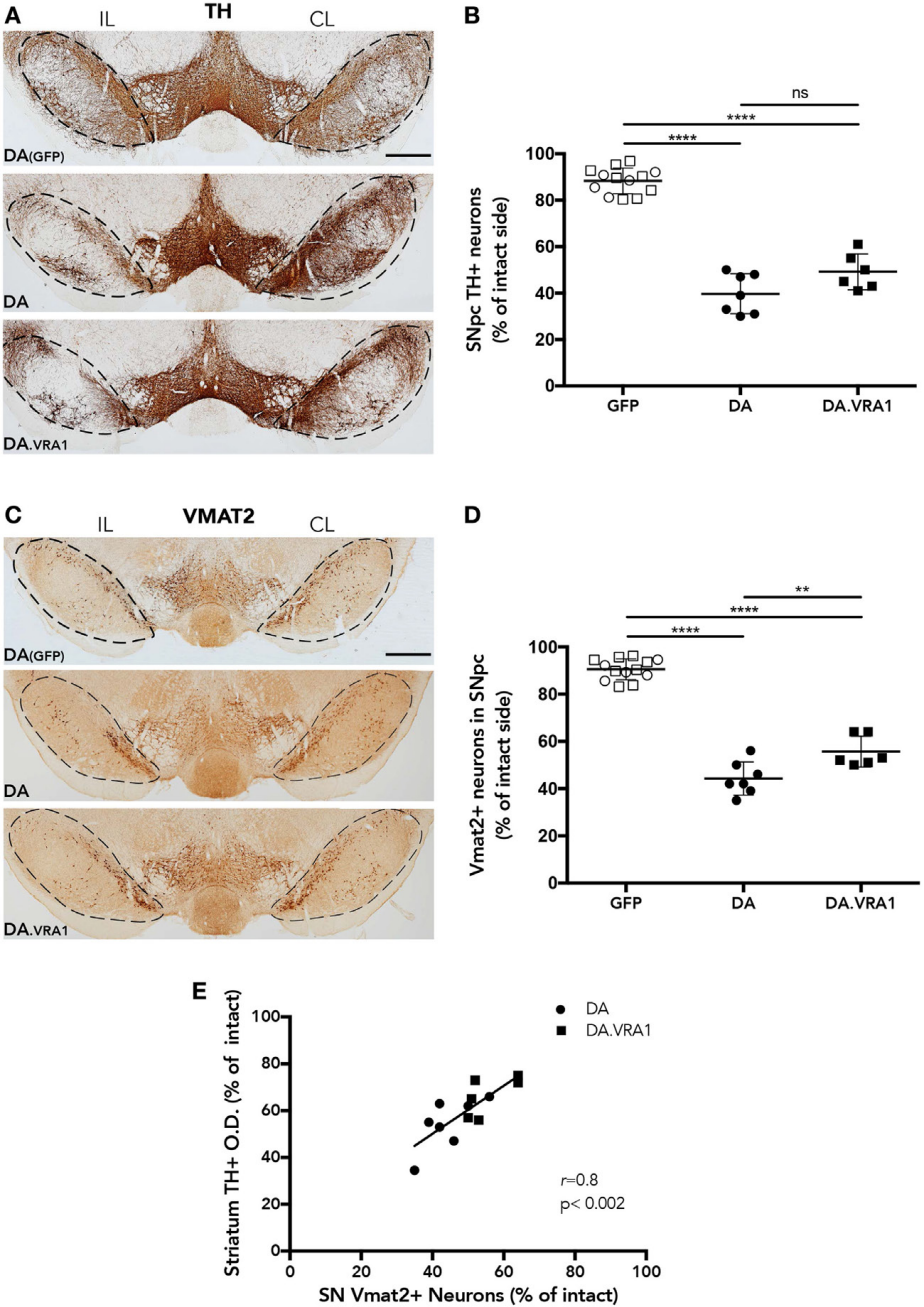
The *Vra1*-locus mediates partial protection of nigral dopaminergic neurons in response to α-syn overexpression. **(A)** Representative images showing midbrain TH^+^ cells in DA(GFP), DA, and DA.VRA1 rats at 8 weeks post unilateral recombinant adeno-associated viral (rAAV)-α-syn injection. Dashed lines represent the area used for stereological cell counts. **(B)** Stereological quantification of TH^+^ neurons in the SN shows no significant difference in the percentage of remaining TH^+^ neurons in the IL side between DA and DA.VRA1. **(C)** Representative images showing midbrain vesicular monoamine transporter 2 (VMAT2)^+^ cells in DA(GFP), DA, and DA.VRA1 rats at 8 weeks post unilateral rAAV-α-syn injection. **(D)** Stereological quantification of VMAT2^+^ dopaminergic neurons at 8 weeks post injection shows a similar pattern as for TH^+^ cells, but with DA.VRA1 congenic rats displaying partial protection to dopaminergic cell loss in the IL substantia nigra pars compacta (SNpc) compared to DA rats. **(E)** The ratio of dopaminergic cells quantified by VMAT2 in the lesioned vs intact SNpc strongly correlates with the relative density of TH^+^ fibers in the dorsal striatum. Individual data points and mean ± SD are shown. CL, contralateral; IL, ipsilateral; scale bars = 500 µm. *p < 0.05, with one-way ANOVA followed by a Bonferroni *post hoc* test. *r* = Pearson correlation coefficient.

Midbrain dopaminergic neurons were quantified at 8 weeks post α-syn overexpression and GFP as a control (Figures 3A–D). Stereological cell counting performed with TH^+^-stained sections shows a reduction in dopaminergic cells in the IL SNpc of both DA and DA.VRA1 congenic rats compared to DA(GFP); however, there was no significant difference in the proportion of remaining TH^+^ neurons between DA and DA.VRA1 rats [50 (9) vs. 40 (8)%, *p* = 0.06, Figure 3B]. Due to the possibility of TH being downregulated, thus giving an underestimation of dopaminergic neurons, VMAT2 was also used as a dopaminergic marker to stain and count nigral cells. VMAT2 is a molecule essential for recruiting cytosolic dopamine into synaptic vescicles, and is, therefore, considered a reliable marker for dopaminergic cells (52, 53). Indeed, when quantifying VMAT2^+^ neurons, we can see Vra1-mediated protection of nigral dopaminergic neurons in the IL SNpc of DA.VRA1 congenic vs DA rats [54 (7) vs. 44 (7)%, *p* < 0.004, Figure 3D]. In order to verify whether the loss of dopaminergic fibers in the striatum reflects the extent of dopaminergic cell death in both strains, we performed a correlation analysis between the two sets of data. We found a strong positive correlation between striatal TH + fiber density and remaining dopaminergic cells in the SNpc marked with VMAT2 in both strains (*p* < 0.002; *r* = 0.8, Figure 3E).

### Gsta4 Is Expressed in Midbrain Astrocytes

**Figure 4:**
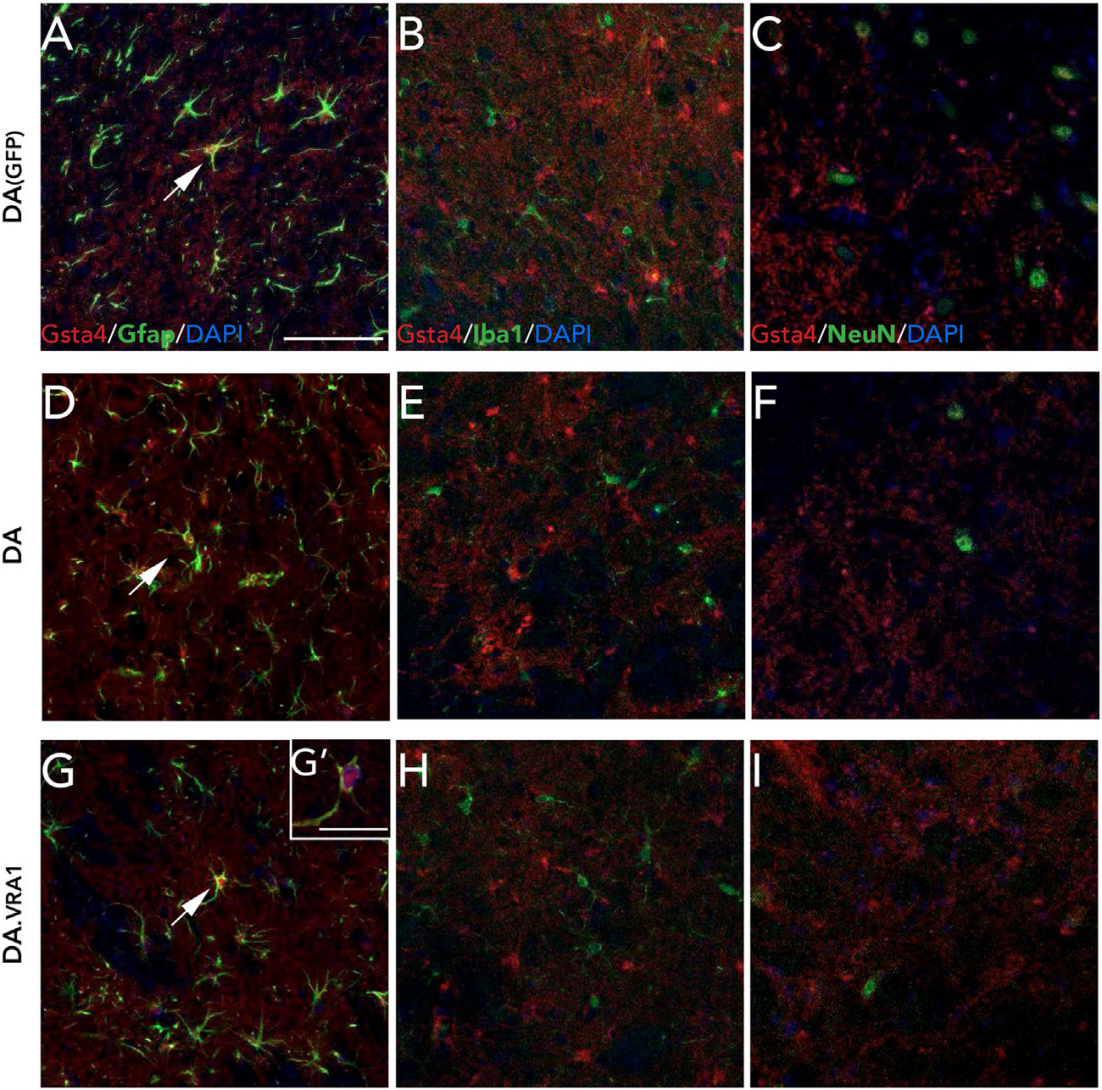
Glutathione *S*-transferase alpha 4 (Gsta4) is expressed in midbrain astrocytes 8 weeks after rAAV-GFP/α-syn injection. Immunofluorescent staining of Gsta4 combined with cell-specific markers for **(A,D,G,G')** astrocytes; Gfap, **(B,E,H)** microglia; Iba1 and **(C,F,I)** neurons; NeuN in DA(GFP), DA, and DA.VRA1 rats. Gsta4 staining co-localized with Gfap **(A,D,G)** but not Iba1 **(B,E,H)** or NeuN **(C,F,I)**, suggesting astrocytic expression. Pictures taken at 20×; scale bar = 20 µm. **(G')** 60× image showing co-localization, with Gsta4 mainly expressed in the soma; scale bar = 100 µm. All markers were combined with the nuclear marker DAPI (blue).

We have previously observed Gsta4 expression in astrocytes but not in microglia or neurons at 8 weeks after 6-OHDA lesion (21). We made the same evaluation with double fluorescence immunostainings on midbrain sections combining Gsta4 with astrocytic (Gfap), microglial (Iba1), or neuronal (NeuN) markers at 8 weeks after α-syn overexpression (Figure 4). The stainings reveal a similar co-localization pattern of Gsta4 with Gfap (Figures 4A,D,G,G**'**) and not Iba1 (Figures 4B,E,H) or NeuN (Figures 4C,F,I) within this model, thus confirming astrocytic expression of Gsta4. This pattern remains constant in DA(GFP), DA, and DA.VRA1 animals (Figures 4A–I). Once again, the co-localization is more clear in the somas of SNpc astrocytes rather than the projections (Figure 4G**'**).

**Figure 5:**
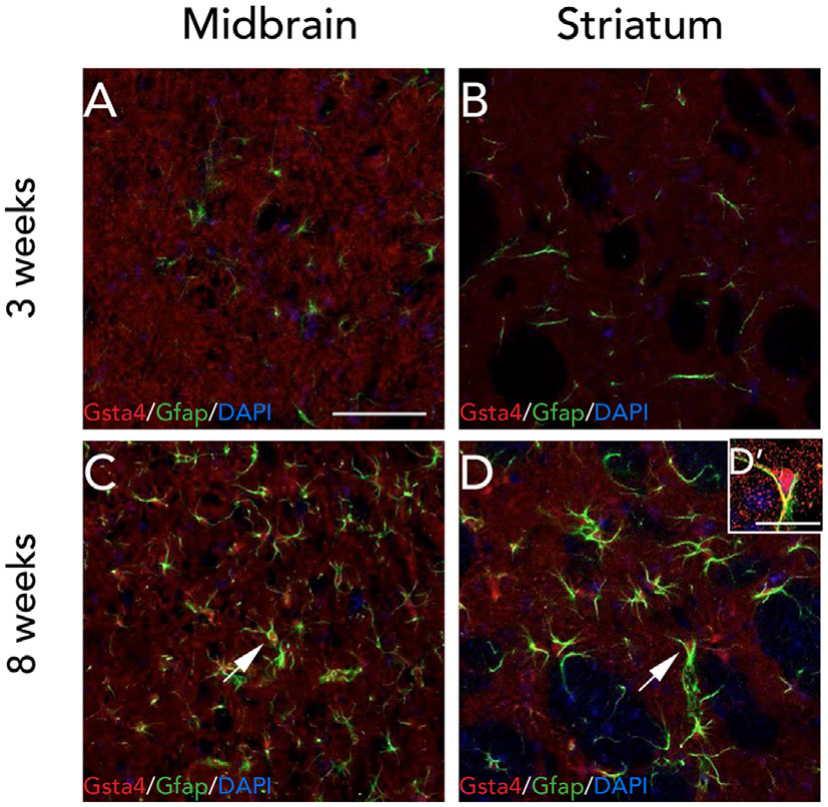
Expression of glutathione S-transferase alpha 4 (Gsta4) and Gfap is increased at 8 weeks after recombinant adeno-associated viral-α-syn injection. Co-immunofluorescent stainings of Gsta4 and Gfap in the midbrain **(A,C)**, and striatum **(B,D)** of a DA.VRA1 congenic rat. Both Gsta4 and Gfap display a lower expression at 3 weeks **(A,B)** compared to 8 weeks **(C,D)**. Pictures taken at 20×, scale bar = 20 µm. **(D')** 60× image showing co-localization of Gsta4 with Gfap; scale bar = 100 µm. Stainings were combined with the nuclear marker DAPI (blue).

Since the gene expression analysis was performed at 3 weeks, and in order to check for any visible differences between Gsta4 gene and protein expression patterns at this time point, we chose to look at Gsta4 localization at 3 weeks as well. Immunofluorescent stainings for Gsta4 and Gfap were compared between midbrain and striatum sections at 3 and 8 weeks post rAAV-α-syn delivery. The staining intensity for both Gsta4 and Gfap is visibly lower at 3 weeks when compared to 8 weeks (Figures 5A–D**'**). At 3 weeks, Gsta4-stained cell bodies do not stand out compared to the background and less Gfap-positive cells are visible. In addition, no co-localization of Gsta4 is detectable with Gfap (Figures 5A,B), NeuN, or Iba1 (data not shown). However, at 8 weeks post rAAV-α-syn delivery, there is clear co-localization of Gsta4 and Gfap in both the midbrain and striatum (Figures 5C,D). This suggests a delayed increase in astrocytic Gsta4 expression in response to α-syn overexpression.

## Discussion

In this study, we show that PVG alleles in the *Vra1* locus partially protect the nigrostriatal pathway of DA.VRA1 congenic rats from α-syn-induced neurodegeneration. At 3 weeks after unilateral rAAV-α-syn delivery, *Gsta4* expression levels were higher in both the IL and CL striatum and midbrain of DA.VRA1 rats compared to DA. When quantifying dopaminergic neurodegeneration at 8 weeks, the density of striatal dopaminergic fibers in the lesioned side was significantly higher in the congenic rats compared to DA, and similar evidence of *Vra1*-mediated neuroprotection was observed for midbrain dopaminergic cell bodies by stereological cell counts of TH^+^ and VMAT2^+^ neurons. These results are in line with our previous observations where DA.VRA1 rats displayed partial dopaminergic neuroprotection to striatal 6-OHDA lesion (21). However, while the toxin-based model results in dopaminergic loss mediated by the generation of ROS and mitochondrial damage, the current study models α-syn-induced pathology, similar to that seen in PD patients. In addition, we show that Gsta4 is expressed in the cytoplasm of midbrain and striatal astrocytes at 8 weeks after α-syn overexpression, suggesting that astrocytes play an important role in protecting nearby neurons and neurites from α-syn-induced toxicity.

Our previous work, detailing the neuroprotective effects of Gsta4 after striatal 6-OHDA injections, puts focus on the oxidative stress aspects of PD. The neurodegenerative process of 6-OHDA is thought to be due to accumulation of ROS (20) and high levels of HNE within the affected cells (18, 54). Furthermore, 6-OHDA models have been shown to reproduce progressive and retrograde degeneration of the nigrostriatal pathway, mirroring some aspects of the same degeneration seen in PD patients with mild to moderate stages of the disease (55, 56). However, the striatal 6-OHDA model does not cover other pathogenic mechanisms of PD, such as the production of toxic α-syn species or impaired protein degradation (57). The functional link between α-syn and PD is very strong, with α-syn-containing Lewy bodies being present in both familial and idiopathic PD, and the SNCA gene being both linked to familial PD and associated to the risk of developing idiopathic PD. The rat rAAV-α-syn model employed here is based on the clear link between α-syn and PD-like pathology and complements the 6-OHDA model, which can be considered a model for toxin-induced PD. The rAAV vector used in the current study includes the WPRE element, which amplifies the expression of the transgene and induces unilateral overexpression of α-syn, progressive dopaminergic neurodegeneration, and motor impairment, which peak at 8 weeks postinjections (42). The rAAV-α-syn model also induces more progressive behavioral impairments compared to the striatal 6-OHDA model, probably due to the buildup of toxic α-syn species leading to deficits in synaptic function (57, 58).

With the striatal 6-OHDA model, aiming to examine causality of the neuroprotection observed in DA.VRA1 rats at 8 weeks, we measured *Gsta4* expression at 2 and 7 days post lesion, when the very early signs of neurodegeneration are seen in the striatum (59). In the rAAV-α-syn model, the first signs of dopaminergic dysfunction and cell loss occur at 3 weeks postinjection (42). Therefore, to keep within the same line of thinking for this study, we performed gene expression analysis of *Gsta4* at 3 weeks. *Gsta4* expression was higher in both striatum and midbrain of DA.VRA1 rats compared to DA rats. The strain difference was seen in both the lesioned and the intact sides, suggesting that α-syn overexpression does not induce *Gsta4* gene expression at this time point. Based on the observation that, along with increased Gfap staining, Gsta4 immunostaining was enhanced at 8 weeks compared to 3 weeks post transgene delivery, there might be a delayed increase in *Gsta4* gene expression after the α-syn transgene overexpression is established. Alternatively, a modest and continuous increase in *Gsta4* gene expression in the DA.VRA1 congenic strain is sufficient to partially protect midbrain dopaminergic cell projections and somas from degeneration.

There is plenty of evidence suggesting that α-syn overexpression increases oxidative stress levels, which is a key feature of PD. Both *in vivo* and *in vitro* models have shown that accumulation of α-syn can lead to mitochondrial dysfunction through the inhibition of Complex 1 (C1), which in turn leads to the production of ROS (60–62). Interestingly, it has also been shown that ROS are a result of depleted glutathione (GSH) in PD brains (63) and low levels of GSH can lead to the decrease of C1 activity (64). Indeed, one important aspect of Gsta4 activity is its ability to catalyze the conjugation of GSH to lipid peroxidation by-products such as HNE (9). Furthermore, a study by Shearn et al. on chronic alcohol consumption in a *Gsta4* null mouse showed that Gsta4 works as a mitochondrial detoxifier (65). This strongly suggests that α-syn toxicity is partly mediated by oxidative stress mechanisms, mainly acting through the mitochondria in dopaminergic cells and involving GSH metabolism. The fact that we see a similar neuroprotective phenotype of DA.VRA1 rats in both the α-syn overexpression model and the striatal 6-OHDA model strongly suggests that the *Vra1* locus encoding Gsta4 regulates key processes in PD-like dopaminergic neurodegeneration. The human ortholog GSTA4 is thus a promising therapeutic target in PD with a complex etiology.

In rat, Gsta proteins have been found to be abundant in astrocytes, the choroid plexus, as well as in endothelial cells and/or astrocytic end feet associated with blood vessels, Purkinje cells, and neurons (66). Therefore, regional differences in the cellular and subcellular distribution of Gsta4 are not unlikely. In our previous work where the *Vra1* locus was found to protect from striatal 6-OHDA lesions, we aimed to uncover the localization of Gsta4 within the affected areas of the rat brain. We found Gsta4 co-expression with the astrocytic marker Gfap, but not with the microglial (Iba1) or the neuronal (NeuN) markers at 8 weeks post injection (21). In the current study, we confirm the astrocytic localization of Gsta4 at 8 weeks in both DA and DA.VRA1 strains. In a nerve injury model, expression of Gsta4 has been shown in spinal motor neurons and not astrocytes (10). Of note, we cannot rule out the possibility that dopaminergic neurons express Gsta4 at levels not detected by immunostainings in our studies.

The relationship between α-syn and astrocytes is well studied. α-syn is found mainly in neurons, but can often accumulate in astrocytes as well, usually after spreading from neurons (67–69), possibly through cell-to-cell transfer (70). A recent study by Lindström et al. points out the important role of astrocytes in α-synucleinopathies. They show that in a co-culture system, astrocytes engulf large amounts of α-syn oligomers but are subsequently not able to degrade them completely, which leads to the formation of inclusions. It suggested that this is most likely brought on by a dysfunctional lysosomal system. Astrocytes also showed signs of mitochondrial damage caused by the accumulation of these α-syn oligomers (71). Furthermore, studies have shown that astrocytes can produce ROS under stressful conditions (72), thus leaving surrounding neurons susceptible to damage (73). This is relevant to the results obtained from DA.VRA1 congenic rats by us (21) and others (10), since ROS production is increased by 6-OHDA (20), α-syn overexpression (61), and in nerve injury models (74)—all environments where DA.VRA1 rats have been shown to express higher levels of *Gsta4*. When adding the fact that astrocytes also have a very high activity and release of GSH, which might be neuroprotective in itself (75), the link between Gsta4 activity and α-syn pathology is strengthened. More work is necessary to uncover the specific mechanisms by which Gsta4 protects from PD-like pathology in rat PD models. For example, a more in-depth analysis of the role of Gsta4 in astrocytic mitochondria might help answer key questions surrounding potential neuroprotective mechanisms.

In conclusion, this is the first report suggesting potential neuroprotective effects of the *Vra1* locus and *Gsta4* in an α-syn-induced PD model. Moreover, this study emphasizes the importance of utilizing animal models with naturally occurring allelic differences in order to gain a better understanding of neurodegenerative diseases with complex traits, such as PD. Gsta4 has now been implicated as a potential neuroprotective agent in both the 6-OHDA and α-syn overexpression PD models, making the human ortholog a very attractive candidate for future PD therapeutic research.

## Ethics Statement

This study was carried out in accordance with the recommendations of the Ethical Committee for the use of laboratory animals in the Lund/Malmö region.

## Author Contributions

MJ and MS conceived and designed the experiments; MJ, ED, KB, MN, and IJ-F performed the experiments; MJ, KB, and ED analyzed the data; MJ and MS wrote the paper with contribution from coauthors.

## Conflict of Interest Statement

The authors declare no conflict of interest. The founding sponsors had no role in the design of the study; in the collection, analyses, or interpretation of data; in the writing of the manuscript, and in the decision to publish the results.

## References

[1] Spillantini MG Crowther RA Jakes R Hasegawa M Goedert M. Alpha-synuclein in filamentous inclusions of Lewy bodies from Parkinson’s disease and dementia with Lewy bodies. Proc Natl Acad Sci U S A (1998) 95(11):6469–73. 10.1073/pnas.95.11.6469 9600990 PMC27806

[2] Lill CM. Genetics of Parkinson’s disease. Mol Cell Probes (2016) 30(6):386–96. 10.1016/j.mcp.2016.11.001 27818248

[3] Ryan SD Dolatabadi N Chan SF Zhang X Akhtar MW Parker J Isogenic human iPSC Parkinson’s model shows nitrosative stress-induced dysfunction in MEF2-PGC1alpha transcription. Cell (2013) 155(6):1351–64. 10.1016/j.cell.2013.11.009 24290359 PMC4028128

[4] Sveinbjornsdottir S The clinical symptoms of Parkinson’s disease. J Neurochem (2016) 139(Suppl 1):318–24. 10.1111/jnc.13691 27401947

[5] Nalls MA Pankratz N Lill CM Do CB Hernandez DG Saad M Large-scale meta-analysis of genome-wide association data identifies six new risk loci for Parkinson’s disease. Nat Genet (2014) 46(9):989–93. 10.1038/ng.3043 25064009 PMC4146673

[6] Chang D Nalls MA Hallgrimsdottir IB Hunkapiller J van der Brug M Cai F A meta-analysis of genome-wide association studies identifies 17 new Parkinson’s disease risk loci. Nat Genet (2017) 49(10):1511–6. 10.1038/ng.3955 28892059 PMC5812477

[7] Lidman O Swanberg M Horvath L Broman KW Olsson T Piehl F. Discrete gene loci regulate neurodegeneration, lymphocyte infiltration, and major histocompatibility complex class II expression in the CNS. J Neurosci (2003) 23(30):9817–23. 14586010 10.1523/JNEUROSCI.23-30-09817.2003 PMC6740878

[8] Swanberg M Harnesk K Strom M Diez M Lidman O Piehl F. Fine mapping of gene regions regulating neurodegeneration. PLoS One (2009) 4(6):e5906. 10.1371/journal.pone.0005906 19526058 PMC2691596

[9] Hubatsch I Ridderstrom M Mannervik B. Human glutathione transferase A4-4: an alpha class enzyme with high catalytic efficiency in the conjugation of 4-hydroxynonenal and other genotoxic products of lipid peroxidation. Biochem J (1998) 330(Pt 1):175–9. 10.1042/bj3300175 9461507 PMC1219124

[10] Strom M Al Nimer F Lindblom R Nyengaard JR Piehl F. Naturally occurring genetic variability in expression of Gsta4 is associated with differential survival of axotomized rat motoneurons. Neuromolecular Med (2012) 14(1):15–29. 10.1007/s12017-011-8164-8 22160604

[11] Al Nimer F Strom M Lindblom R Aeinehband S Bellander BM Nyengaard JR Naturally occurring variation in the glutathione-S-transferase 4 gene determines neurodegeneration after traumatic brain injury. Antioxid Redox Signal (2013) 18(7):784–94. 10.1089/ars.2011.4440 22881716 PMC3555113

[12] Martinez-Lara E Siles E Hernandez R Canuelo AR Luisa del Moral M Jimenez A Glutathione S-transferase isoenzymatic response to aging in rat cerebral cortex and cerebellum. Neurobiol Aging (2003) 24(3):501–9. 10.1016/S0197-4580(02)00139-2 12600725

[13] Singhal SS Zimniak P Sharma R Srivastava SK Awasthi S Awasthi YC. A novel glutathione S-transferase isozyme similar to GST 8-8 of rat and mGSTA4-4 (GST 5.7) of mouse is selectively expressed in human tissues. Biochim Biophys Acta (1994) 1204(2):279–86. 10.1016/0167-4838(94)90019-1 8142470

[14] Zimniak P Singhal SS Srivastava SK Awasthi S Sharma R Hayden JB Estimation of genomic complexity, heterologous expression, and enzymatic characterization of mouse glutathione S-transferase mGSTA4-4 (GST 5.7). J Biol Chem (1994) 269(2):992–1000. 7904605

[15] Qian J Jing J Jin G Wang H Wang Y Liu H Association between polymorphisms in the GSTA4 gene and risk of lung cancer: a case-control study in a Southeastern Chinese population. Mol Carcinog (2009) 48(3):253–9. 10.1002/mc.20478 18767114

[16] Li CG Zhao ZM Hu MG Liu R. Predictive role of glutathione-S-transferase gene polymorphisms in risk and prognosis of hepatocellular carcinoma. Asian Pac J Cancer Prev (2012) 13(7):3247–52. 10.7314/APJCP.2012.13.7.3247 22994742

[17] Yoritaka A Hattori N Uchida K Tanaka M Stadtman ER Mizuno Y. Immunohistochemical detection of 4-hydroxynonenal protein adducts in Parkinson disease. Proc Natl Acad Sci U S A (1996) 93(7):2696–701. 10.1073/pnas.93.7.2696 8610103 PMC39693

[18] Selley ML. (E)-4-hydroxy-2-nonenal may be involved in the pathogenesis of Parkinson’s disease. Free Radic Biol Med (1998) 25(2):169–74. 10.1016/S0891-5849(98)00021-5 9667492

[19] Castellani RJ Perry G Siedlak SL Nunomura A Shimohama S Zhang J Hydroxynonenal adducts indicate a role for lipid peroxidation in neocortical and brainstem Lewy bodies in humans. Neurosci Lett (2002) 319(1):25–8. 10.1016/S0304-3940(01)02514-9 11814645

[20] Glinka Y Gassen M Youdim MB. Mechanism of 6-hydroxydopamine neurotoxicity. J Neural Transm Suppl (1997) 50:55–66. 10.1007/978-3-7091-6842-4_7 9120425

[21] Jewett M Jimenez-Ferrer I Swanberg M. Astrocytic expression of GSTA4 is associated to dopaminergic neuroprotection in a rat 6-OHDA model of Parkinson’s disease. Brain Sci (2017) 7(7):E73. 10.3390/brainsci7070073 28672859 PMC5532586

[22] Klein C Westenberger A Genetics of Parkinson’s disease. Cold Spring Harb Perspect Med (2012) 2(1):a008888 10.1101/cshperspect.a008888 22315721 PMC3253033

[23] Chartier-Harlin MC Kachergus J Roumier C Mouroux V Douay X Lincoln S Alpha-synuclein locus duplication as a cause of familial Parkinson’s disease. Lancet (2004) 364(9440):1167–9. 10.1016/s0140-6736(04)17103-1 15451224

[24] Ibanez P Bonnet AM Debarges B Lohmann E Tison F Pollak P Causal relation between alpha-synuclein gene duplication and familial Parkinson’s disease. Lancet (2004) 364(9440):1169–71. 10.1016/s0140-6736(04)17104-3 15451225

[25] Nishioka K Hayashi S Farrer MJ Singleton AB Yoshino H Imai H Clinical heterogeneity of alpha-synuclein gene duplication in Parkinson’s disease. Ann Neurol (2006) 59(2):298–309. 10.1002/ana.20753 16358335

[26] Fuchs J Nilsson C Kachergus J Munz M Larsson EM Schule B Phenotypic variation in a large Swedish pedigree due to SNCA duplication and triplication. Neurology (2007) 68(12):916–22. 10.1212/01.wnl.0000254458.17630.c5 17251522

[27] Ahn TB Kim SY Kim JY Park SS Lee DS Min HJ Alpha-synuclein gene duplication is present in sporadic Parkinson disease. Neurology (2008) 70(1):43–9. 10.1212/01.wnl.0000271080.53272.c7 17625105

[28] Brueggemann N Odin P Gruenewald A Tadic V Hagenah J Seidel G Re: alpha-synuclein gene duplication is present in sporadic Parkinson disease. Neurology (2008) 71(16):1294; author reply 1294 10.1212/01.wnl.0000338439.00992.c7 18852448

[29] Ikeuchi T Kakita A Shiga A Kasuga K Kaneko H Tan CF Patients homozygous and heterozygous for SNCA duplication in a family with parkinsonism and dementia. Arch Neurol (2008) 65(4):514–9. 10.1001/archneur.65.4.514 18413475

[30] Troiano AR Cazeneuve C Le Ber I Bonnet AM Lesage S Brice A Re: alpha-synuclein gene duplication is present in sporadic Parkinson disease. Neurology (2008) 71(16):1295; author reply 1295 10.1212/01.wnl.0000338435.78120.0f 18852449

[31] Uchiyama T Ikeuchi T Ouchi Y Sakamoto M Kasuga K Shiga A Prominent psychiatric symptoms and glucose hypometabolism in a family with a SNCA duplication. Neurology (2008) 71(16):1289–91. 10.1212/01.wnl.0000327607.28928.e6 18852445

[32] Ibanez P Lesage S Janin S Lohmann E Durif F Destee A Alpha-synuclein gene rearrangements in dominantly inherited parkinsonism: frequency, phenotype, and mechanisms. Arch Neurol (2009) 66(1):102–8. 10.1001/archneurol.2008.555 19139307

[33] Singleton AB Farrer M Johnson J Singleton A Hague S Kachergus J Alpha-synuclein locus triplication causes Parkinson’s disease. Science (2003) 302(5646):841 10.1126/science.1090278 14593171

[34] Farrer M Kachergus J Forno L Lincoln S Wang DS Hulihan M Comparison of kindreds with parkinsonism and alpha-synuclein genomic multiplications. Ann Neurol (2004) 55(2):174–9. 10.1002/ana.10846 14755720

[35] Simon-Sanchez J Schulte C Bras JM Sharma M Gibbs JR Berg D Genome-wide association study reveals genetic risk underlying Parkinson’s disease. Nat Genet (2009) 41(12):1308–12. 10.1038/ng.487 19915575 PMC2787725

[36] Harvey BK Wang Y Hoffer BJ. Transgenic rodent models of Parkinson’s disease. Acta Neurochir Suppl (2008) 101:89–92. 10.1007/978-3-211-78205-7_15 18642640 PMC2613245

[37] Blesa J Przedborski S. Parkinson’s disease: animal models and dopaminergic cell vulnerability. Front Neuroanat (2014) 8:155. 10.3389/fnana.2014.00155 25565980 PMC4266040

[38] Visanji NP Brotchie JM Kalia LV Koprich JB Tandon A Watts JC Alpha-synuclein-based animal models of Parkinson’s disease: challenges and opportunities in a new era. Trends Neurosci (2016) 39(11):750–62. 10.1016/j.tins.2016.09.003 27776749

[39] Kirik D Rosenblad C Burger C Lundberg C Johansen TE Muzyczka N Parkinson-like neurodegeneration induced by targeted overexpression of alpha-synuclein in the nigrostriatal system. J Neurosci (2002) 22(7):2780–91. 11923443 10.1523/JNEUROSCI.22-07-02780.2002 PMC6758323

[40] Kirik D Annett LE Burger C Muzyczka N Mandel RJ Bjorklund A. Nigrostriatal alpha-synucleinopathy induced by viral vector-mediated overexpression of human alpha-synuclein: a new primate model of Parkinson’s disease. Proc Natl Acad Sci U S A (2003) 100(5):2884–9. 10.1073/pnas.0536383100 12601150 PMC151435

[41] Azeredo da Silveira S Schneider BL Cifuentes-Diaz C Sage D Abbas-Terki T Iwatsubo T Phosphorylation does not prompt, nor prevent, the formation of alpha-synuclein toxic species in a rat model of Parkinson’s disease. Hum Mol Genet (2009) 18(5):872–87. 10.1093/hmg/ddn417 19074459

[42] Decressac M Mattsson B Lundblad M Weikop P Bjorklund A Progressive neurodegenerative and behavioural changes induced by AAV-mediated overexpression of alpha-synuclein in midbrain dopamine neurons. Neurobiol Dis (2012) 45(3):939–53. 10.1016/j.nbd.2011.12.013 22182688

[43] Febbraro F Sahin G Farran A Soares S Jensen PH Kirik D Ser129D mutant alpha-synuclein induces earlier motor dysfunction while S129A results in distinctive pathology in a rat model of Parkinson’s disease. Neurobiol Dis (2013) 56:47–58. 10.1016/j.nbd.2013.03.014 23567651

[44] Hsu LJ Sagara Y Arroyo A Rockenstein E Sisk A Mallory M α-Synuclein promotes mitochondrial deficit and oxidative stress. Am J Pathol (2000) 157(2):401–10. 10.1016/S0002-9440(10)64553-1 10934145 PMC1850140

[45] Esteves AR Arduino DM Swerdlow RH Oliveira CR Cardoso SM. Oxidative stress involvement in alpha-synuclein oligomerization in Parkinson’s disease cybrids. Antioxid Redox Signal (2009) 11(3):439–48. 10.1089/ars.2008.2247 18717628

[46] Rocha EM De Miranda B Sanders LH Alpha-synuclein: pathology, mitochondrial dysfunction and neuroinflammation in Parkinson’s disease. Neurobiol Dis (2017) 109(Pt B):249–57. 10.1016/j.nbd.2017.04.004 28400134

[47] Tapias V Hu X Luk KC Sanders LH Lee VM Greenamyre JT. Synthetic alpha-synuclein fibrils cause mitochondrial impairment and selective dopamine neurodegeneration in part via iNOS-mediated nitric oxide production. Cell Mol Life Sci (2017) 74(15):2851–74. 10.1007/s00018-017-2541-x 28534083 PMC5524146

[48] George Paxinos CW The Rat Brain in Stereotaxic Coordinates. San Diego: Academic Press (2007).

[49] Gundersen HJ Jensen EB Kieu K Nielsen J The efficiency of systematic sampling in stereology – reconsidered. J Microsc (1999) 193(Pt 3):199–211. 10.1046/j.1365-2818.1999.00457.x 10348656

[50] Livak Schmittgen Analysis of relative gene expression data using real-time quantitative PCR and the 2(-Delta Delta C(T)) Method. Methods (2001) 25(4), 402–408. 10.1006/meth.2001.1262 11846609

[51] Voorn P Vanderschuren LJ Groenewegen HJ Robbins TW Pennartz CM. Putting a spin on the dorsal-ventral divide of the striatum. Trends Neurosci (2004) 27(8):468–74. 10.1016/j.tins.2004.06.006 15271494

[52] Takahashi N Miner LL Sora I Ujike H Revay RS Kostic V VMAT2 knockout mice: heterozygotes display reduced amphetamine-conditioned reward, enhanced amphetamine locomotion, and enhanced MPTP toxicity. Proc Natl Acad Sci U S A (1997) 94(18):9938–43. 10.1073/pnas.94.18.9938 9275230 PMC23302

[53] Miller GW Erickson JD Perez JT Penland SN Mash DC Rye DB Immunochemical analysis of vesicular monoamine transporter (VMAT2) protein in Parkinson’s disease. Exp Neurol (1999) 156(1):138–48. 10.1006/exnr.1998.7008 10192785

[54] Smith MP Cass WA. Oxidative stress and dopamine depletion in an intrastriatal 6-hydroxydopamine model of Parkinson’s disease. Neuroscience (2007) 144(3):1057–66. 10.1016/j.neuroscience.2006.10.004 17110046 PMC2048571

[55] Kirik D Rosenblad C Bjorklund A. Characterization of behavioral and neurodegenerative changes following partial lesions of the nigrostriatal dopamine system induced by intrastriatal 6-hydroxydopamine in the rat. Exp Neurol (1998) 152(2):259–77. 10.1006/exnr.1998.6848 9710526

[56] Deumens R Blokland A Prickaerts J. Modeling Parkinson’s disease in rats: an evaluation of 6-OHDA lesions of the nigrostriatal pathway. Exp Neurol (2002) 175(2):303–17. 10.1006/exnr.2002.7891 12061862

[57] Venda LL Cragg SJ Buchman VL Wade-Martins R Alpha-synuclein and dopamine at the crossroads of Parkinson’s disease. Trends Neurosci (2010) 33(12):559–68. 10.1016/j.tins.2010.09.004 20961626 PMC3631137

[58] Decressac M Mattsson B Bjorklund A Comparison of the behavioural and histological characteristics of the 6-OHDA and alpha-synuclein rat models of Parkinson’s disease. Exp Neurol (2012) 235(1):306–15. 10.1016/j.expneurol.2012.02.012 22394547

[59] Rosenblad C Kirik D Bjorklund A. Sequential administration of GDNF into the substantia nigra and striatum promotes dopamine neuron survival and axonal sprouting but not striatal reinnervation or functional recovery in the partial 6-OHDA lesion model. Exp Neurol (2000) 161(2):503–16. 10.1006/exnr.1999.7296 10686072

[60] Junn E Mouradian MM. Human alpha-synuclein over-expression increases intracellular reactive oxygen species levels and susceptibility to dopamine. Neurosci Lett (2002) 320(3):146–50. 10.1016/S0304-3940(02)00016-2 11852183

[61] Vali S Chinta SJ Peng J Sultana Z Singh N Sharma P Insights into the effects of alpha-synuclein expression and proteasome inhibition on glutathione metabolism through a dynamic in silico model of Parkinson’s disease: validation by cell culture data. Free Radic Biol Med (2008) 45(9):1290–301. 10.1016/j.freeradbiomed.2008.08.002 18761401 PMC2744580

[62] Moon HE Paek SH. Mitochondrial dysfunction in Parkinson’s disease. Exp Neurobiol (2015) 24(2):103–16. 10.5607/en.2015.24.2.103 26113789 PMC4479806

[63] Jenner P Olanow CW. The pathogenesis of cell death in Parkinson’s disease. Neurology (2006) 66(10 Suppl 4):S24–36. 10.1212/WNL.66.10_suppl_4.S24 16717250

[64] Dias V Junn E Mouradian MM. The role of oxidative stress in Parkinson’s disease. J Parkinsons Dis (2013) 3(4):461–91. 10.3233/jpd-130230 24252804 PMC4135313

[65] Shearn CT Fritz KS Shearn AH Saba LM Mercer KE Engi B Deletion of GSTA4-4 results in increased mitochondrial post-translational modification of proteins by reactive aldehydes following chronic ethanol consumption in mice. Redox Biol (2016) 7:68–77. 10.1016/j.redox.2015.11.013 26654979 PMC4683459

[66] Johnson JA el Barbary A Kornguth SE Brugge JF Siegel FL Glutathione S-transferase isoenzymes in rat brain neurons and glia. J Neurosci (1993) 13(5):2013–23. 8478688 10.1523/JNEUROSCI.13-05-02013.1993 PMC6576553

[67] Croisier E Graeber MB. Glial degeneration and reactive gliosis in alpha-synucleinopathies: the emerging concept of primary gliodegeneration. Acta Neuropathol (2006) 112(5):517–30. 10.1007/s00401-006-0119-z 16896905

[68] Braak H Sastre M Del Tredici K. Development of alpha-synuclein immunoreactive astrocytes in the forebrain parallels stages of intraneuronal pathology in sporadic Parkinson’s disease. Acta Neuropathol (2007) 114(3):231–41. 10.1007/s00401-007-0244-3 17576580

[69] Fellner L Stefanova N The role of glia in alpha-synucleinopathies. Mol Neurobiol (2013) 47(2):575–86. 10.1007/s12035-012-8340-3 22941028 PMC3589649

[70] Angot E Steiner JA Lema Tome CM Ekstrom P Mattsson B Bjorklund A Alpha-synuclein cell-to-cell transfer and seeding in grafted dopaminergic neurons in vivo. PLoS One (2012) 7(6):e39465. 10.1371/journal.pone.0039465 22737239 PMC3380846

[71] Lindstrom Extensive uptake of alpha-synuclein oligomers in astrocytes results in sustained intracellular deposits and mitochondrial damage. Mol Cell Neurosci, (2017) 82:143–156. 10.1016/j.mcn.2017.04.009 28450268

[72] Sheng WS Hu S Feng A Rock RB. Reactive oxygen species from human astrocytes induced functional impairment and oxidative damage. Neurochem Res (2013) 38(10):2148–59. 10.1007/s11064-013-1123-z 23918204 PMC3798006

[73] Sugiyama K Brunori A Mayer ML. Glial uptake of excitatory amino acids influences neuronal survival in cultures of mouse hippocampus. Neuroscience (1989) 32(3):779–91. 10.1016/0306-4522(89)90298-4 2574833

[74] Lieven CJ Hoegger MJ Schlieve CR Levin LA. Retinal ganglion cell axotomy induces an increase in intracellular superoxide anion. Invest Ophthalmol Vis Sci (2006) 47(4):1477–85. 10.1167/iovs.05-0921 16565382

[75] Heales SJ Lam AA Duncan AJ Land JM. Neurodegeneration or neuroprotection: the pivotal role of astrocytes. Neurochem Res (2004) 29(3):513–9. 10.1023/B:NERE.0000014822.69384.0f 15038599

